# Percutaneous & Mini Invasive Achilles tendon repair

**DOI:** 10.1186/1758-2555-3-28

**Published:** 2011-11-14

**Authors:** Michael R Carmont, Roberto Rossi, Sven Scheffler, Omer Mei-Dan, Philippe Beaufils

**Affiliations:** 1 Department of Orthopaedic Surgery, Princess Royal Hospital, Telford, UK; 2 Department of Orthopaedic Surgery, Mauriziano Umberto I Hospital, University of Torino, Italy; 3 Department of Sport Surgery, Centre for Musculoskeletal Surgery, Charite Universitatsmedizin, Berlin, Germany; 4 Department of Sports Medicine, Meir University Hospital, Kfar-Saba, Israel; 5 Andre Mignot Hospital, Versailles Saint Quentin University, F 78150 Le Chesnay, France

## Abstract

Rupture of the Achilles tendon is a considerable cause of morbidity with reduced function following injury. Recent studies have shown little difference in outcome between the techniques of open and non-operative treatment using an early active rehabilitation programme. Meta-analyses have shown that non-operative management has increased risk of re-rupture whereas surgical intervention has risks of complications related to the wound and iatrogenic nerve injury. Minimally invasive surgery has been adopted as a way of reducing infections rates and wound breakdown however avoiding iatrogenic nerve injury must be considered. We discuss the techniques and outcomes of percutaneous and minimally invasive repairs of the Achilles tendon.

## Introduction

Rupture of the Achilles tendon has an incidence of 18 per 100000 [1] and has been shown to be increasing [2]. Achilles tendon rupture has been shown to cause significant morbidity and regardless of treatment major functional deficits persist 2 years after acute Achilles tendon rupture [3] and only 50-60% of elite sportsmen return to pre-injury levels following rupture [4].

Treatment options consist of non-operative management using a cast or functional bracing, percutaneous, minimally invasive and open repair, with or without augmentation.

Recent prospective randomised controlled studies have failed to show a significant difference in outcome between non-operative treatment and surgical repair [5,6]. A meta-analysis has revealed that there is a lower re-rupture rate with open operative treatment (RR 0.27) however surgical intervention carries the risk of infection, wound breakdown and iatrogenic nerve injury (1.88) [7,8].

Clearly treatment must be tailored to the needs of each individual patient. We discuss the use of minimally invasive and percutaneous surgery, including the comparative outcomes, risks and methods to avoid iatrogenic nerve injury.

Commonly used functional outcome scores include the AOFAS [9], the Leppilahti score [10], Merkels scale [11] and the recently developed validated Achilles tendon Total Rupture Score [12]. The risk of tendon re-rupture must also be considered in addition to the complications of surgery, infection, wound breakdown and nerve injury.

Deep venous thrombosis rates have been shown to be similar whether the tendon is repaired of managed non-operatively [13] although increased susceptibility may be considered when these occur following surgery. Rates of DVT following repair has been considered to be similar to that of total hip arthroplasty [14] and it has been recommended that chemical DVT prophylaxis be used.

**Figure 1:**
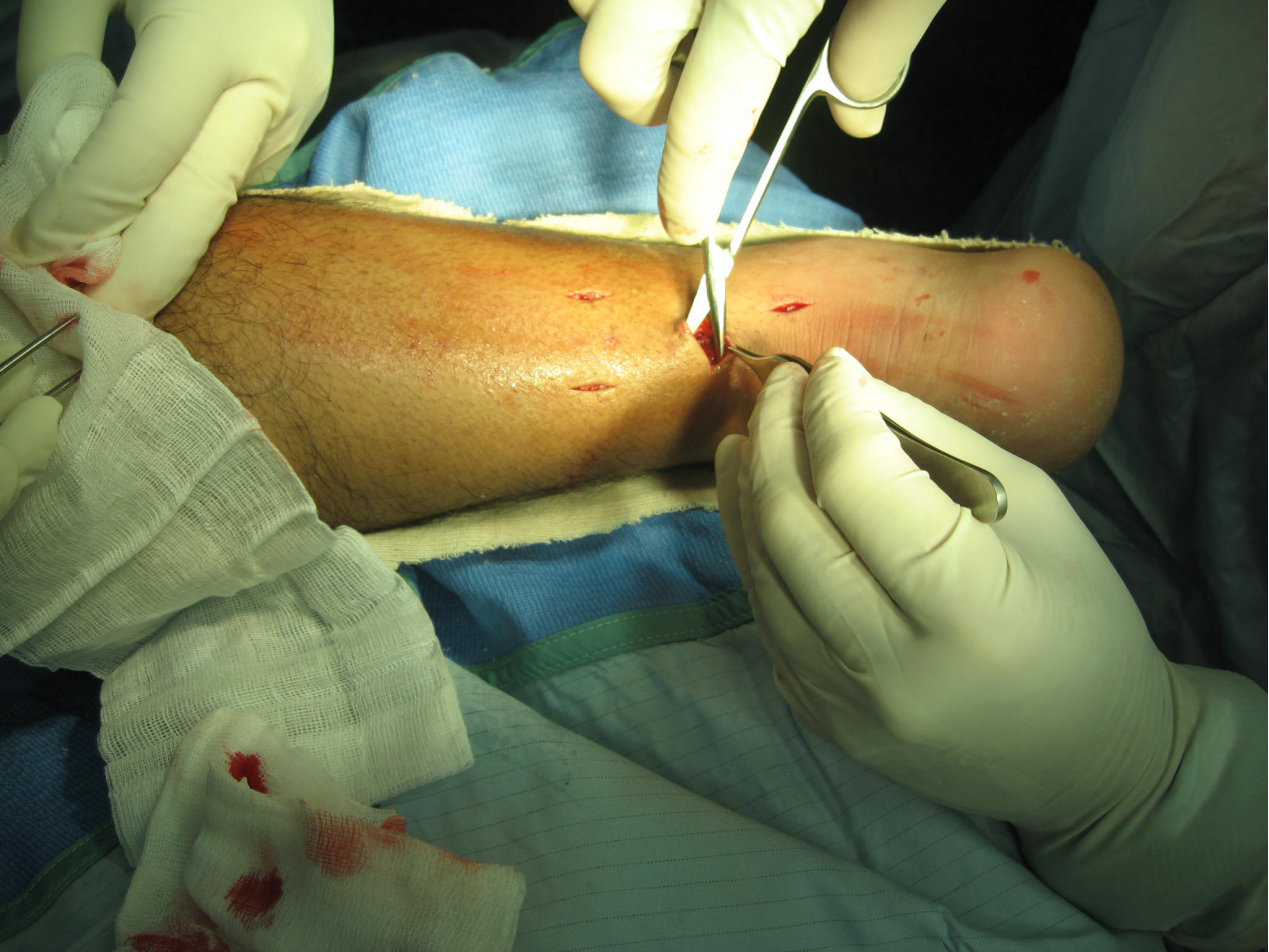
**Visualisation of the sural nerve by careful dissection during minimally invasive Achilles repair**.

The anatomical course of the sural nerve has been studied extensively [15-18] and the incisions utilised to insert this suture technique have been adapted so that the nerve can be visualised [19] (Figure 1) or avoided with a degree of confidence [20]. Knowledge of the anatomy of the sural nerve has been based upon cadaveric studies and in one case using computer assisted determination from slices taken in transverse and sagittal planes. Citak stated that the nerve crossed 11 cm proximal to the tuber calcanei [21].

Although percutaneous repair of the Achilles tendon is known to minimise adhesions, adhesions can lead to ongoing symptoms related to the Achilles tendon and this can be misdiagnosed as a re-rupture [22].

Given the importance of economy related to the provision of health care the actual cost of treatment must also be considered. Percutaneous repair under local anaesthetic has been estimated to be one third of the cost of open surgery [23].

Several review articles have been written on percutaneous and minimally invasive surgery [24-27] and this article presents the current literature on the subject and published evidence.

### Types & Outcomes of percutaneous and minimally invasive techniques: Case series

**Figure 2:**
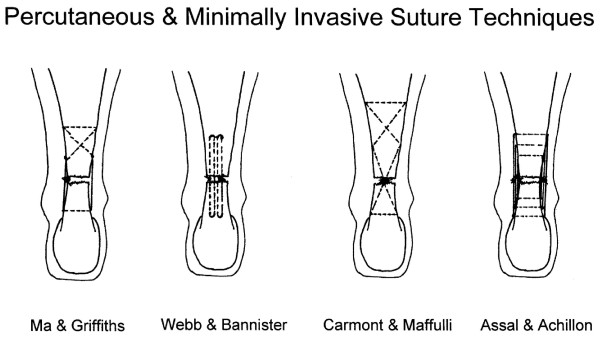
**Suture configurations during Achilles tendon repair: Ma & Griffiths, Webb & Bannister, Carmont & Maffulli and the box suture produced using the Achillon jig**.

A review of the literature has failed to find definitions of the terms percutanous and minimally invasive repair. It would be reasonable to distinguish the two according to the visualisation of apposition of the tendon ends during the repair process. With minimally invasive repair, the tendon ends can be seen apposed through the small incision although the sutures themselves may be passed through stab incisions either side of the tendon. By contrast using a percutaneous repair the tendon ends may not be seen directly but may be visualised using ultrasound and endoscopy. The resting tone of the ankle and a calf squeeze test can indicate the restoration of the tenodesis effect of the repair in both cases. A number of different suture configurations have been used to perform Percutaneous and Minimally Invasive repairs (Figure 2).

The first percutaneous technique was described by Ma & Griffith in 1977 [28]. This technique developed a reputation for iatrogenic sural nerve injuries, with Klein reporting a 13% rate of sural nerve involvement [29], although is still commonly used. Klein recommends the use of absorbable sutures to reduce the symptoms should nerve injury occur. The Ma & Griffith repair consists of a Bunnel suture applied to the proximal tendon and a box suture distally in the stump inserted through 6 para-tendinous stab incisions. Ma & Griffith reported no nerve injuries themselves and good functional outcome. Rouvillain recently reports a series of 60 repairs using this technique, without sural nerve lesion, 2 re-ruptures at 2 and 5 months respectively and mean return to work of 85 days and return to sports in 5 months [30].

In 1992 Delponte described a new technique treated with Tenolig (Fournitures Hospitalieres Industrie, Quimper, France) [31], using a harpoon wire. Independent reports have shown values of 6.5% having sural nerve injuries. The re-rupture rate was similar to that of non-operative treatment (10%). In light of these complication rates Maes' group have changed their technique to mini open technique [32].

Webb and Bannister developed a percutaneous technique, placing the sutures in the midline rather than the side close to the nerve to avoid the risk of iatrogenic injury. Two nylon box stitches were inserted through three stab incisions [33]. Their series of 27 patients were reviewed at 35 months post injury. They returned to work at 4 weeks and sports activities at 4 months. There were no sural nerve injuries or late re-ruptures.

Dresden instruments, using a pDi suture, has been used in a series of 61 patients. 39 patients from this series were reviewed with the majority of patients reporting good or very good outcome, there were no sural nerve injuries and a re-rupture rate of 3.2% [34].

Two Lengemann extension wires were used for co-adaptation of the ruptured tendon together with fibrin sealant at the rupture site. Outcomes were described as very good in 98%. One patient suffered a re-rupture [35]. Similarly Dacron threads applied to malleable needles and a harpoon were used in Martinelli's series reporting return to pre-injury sports activity after 120 to 150 days (3-5 months) [36]. Ng recommended a double-ended needle technique using a standard mini Steinmann pin. In their series there was one sural nerve injury and one late re-rupture in an open repair group. There was less calf atrophy shorter hospital stay and less calf atrophy in those having minimally invasive surgery [37]. A Bunnel type suture has been inserted through a medial skin incision in a series of 14 patients. There were no cases of re-rupture or sural nerve injury and returned to work in 6 weeks [38].

**Figure 3:**
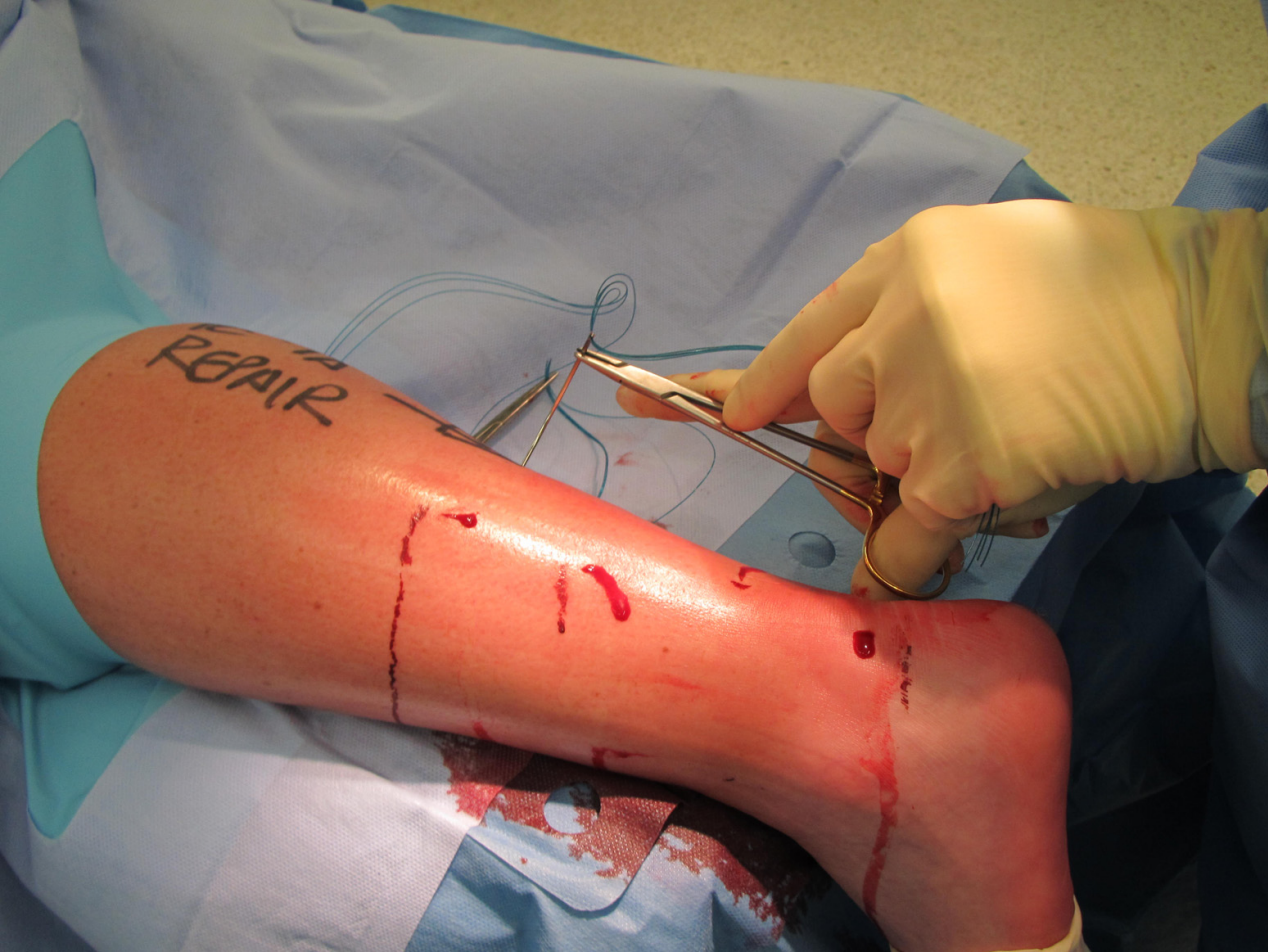
**Percutaneous technique performed under local anaesthesia, note the lack of tourniquet**.

**Figure 4:**
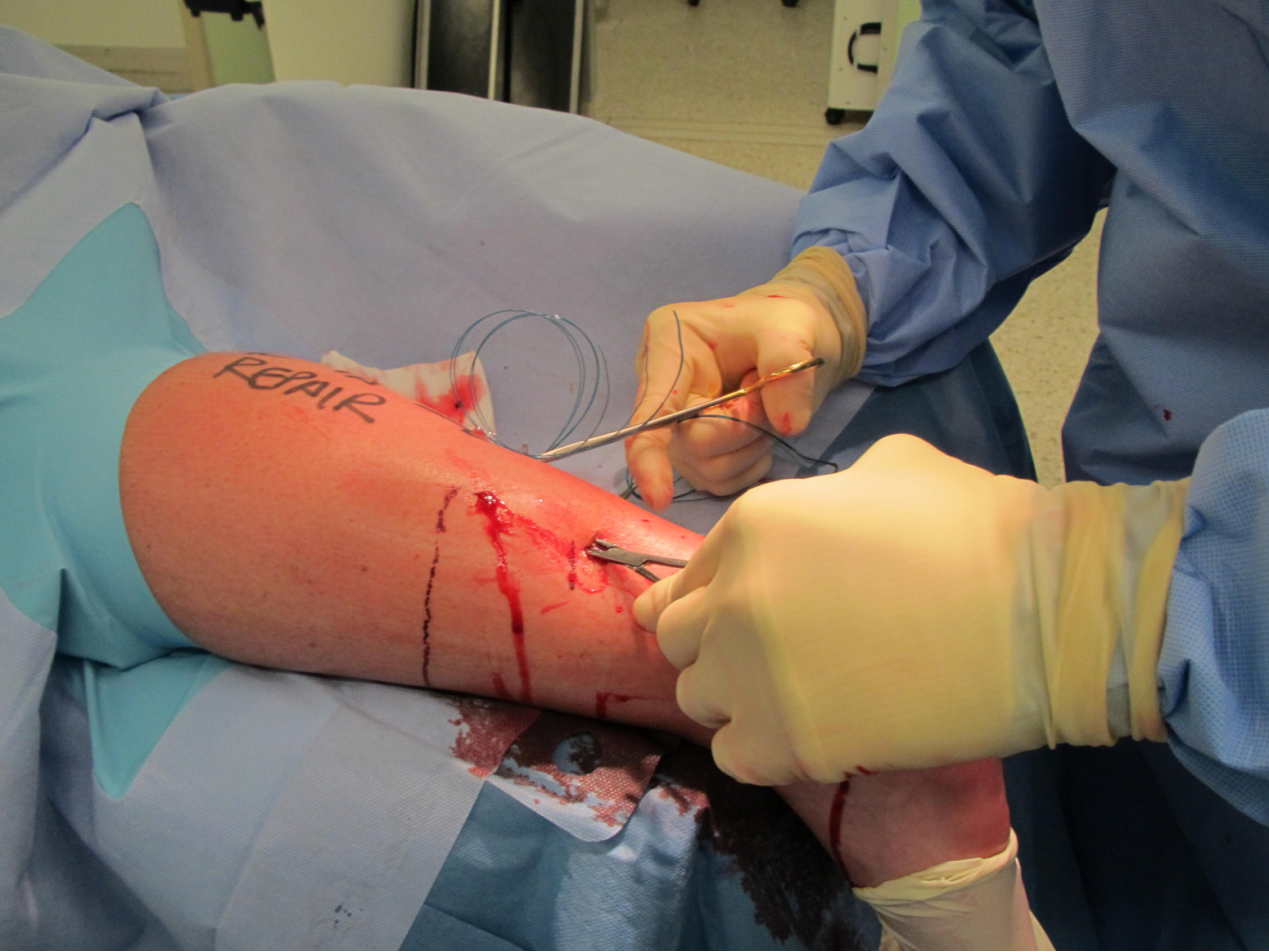
**The use of a nick and spread method at the region where the surl nerve crosses the lateral border of the Achilles tendon**.

McClelland described another modification of Ma & Griffiths' technique [19] and this was subsequently modified [20]. The current version uses an 8 strand repair of Number 1 Maxon inserted using a 9 cm Mayo needle (BL059N, B00 round point spring eye. B Braun Aesculap, Tuttlingen, Germany). The needle is passed through stab paratendinous incisions in a Bunnel fashion emerging through a central stab at the rupture site (Figure 3). The large radius of curvature of the needle means that the stab incisions tend to avoid the path of the sural nerve [20]. The use of a clip in the nick and spread fashion may also prevent nerve damage during suture placement (Figure 4).

Maffulli and co-workers have reported the outcome of this technique in both elderly patients and elite athletes. In 35 patients all over 65 years of age, mean 73.4 years the post operative ATRS was 69.4 and all patients were able to fully weight bear on the affected leg by the 8^th ^post-operative week [39]. Elite athletes were retrospectively assessed at an average of 72 months from the procedure. All patients reported that they were able to weight bare fully at the end of the 8^th ^post-operative week and that the average time to return to sport was 4.8 months. Notably 2 out of the 15 patients in this series reported superficial wound infections [40].

**Figure 5:**
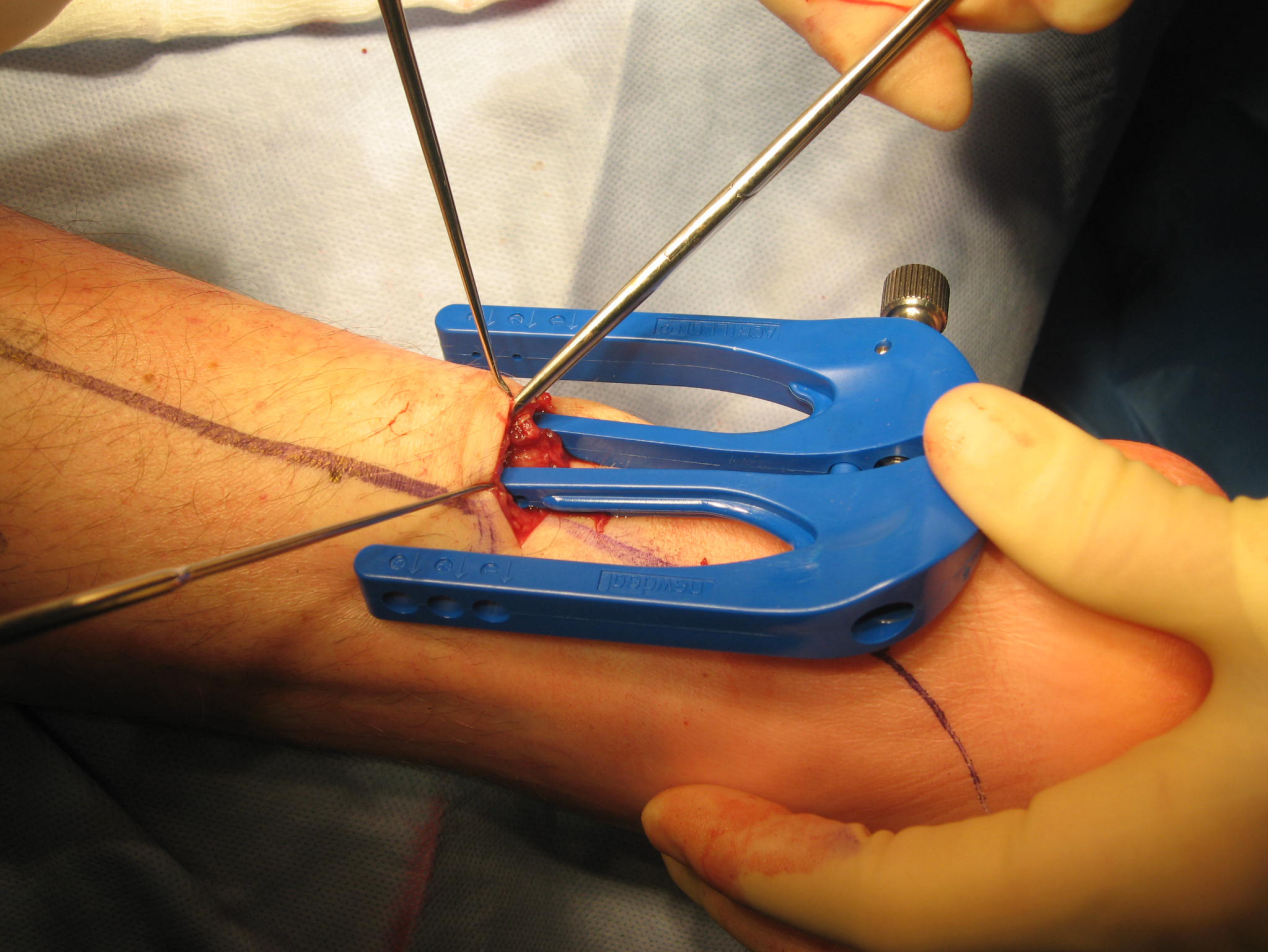
**The Achillon jig being inserted**. The central two branches passing beneath the paratenon.

**Figure 6:**
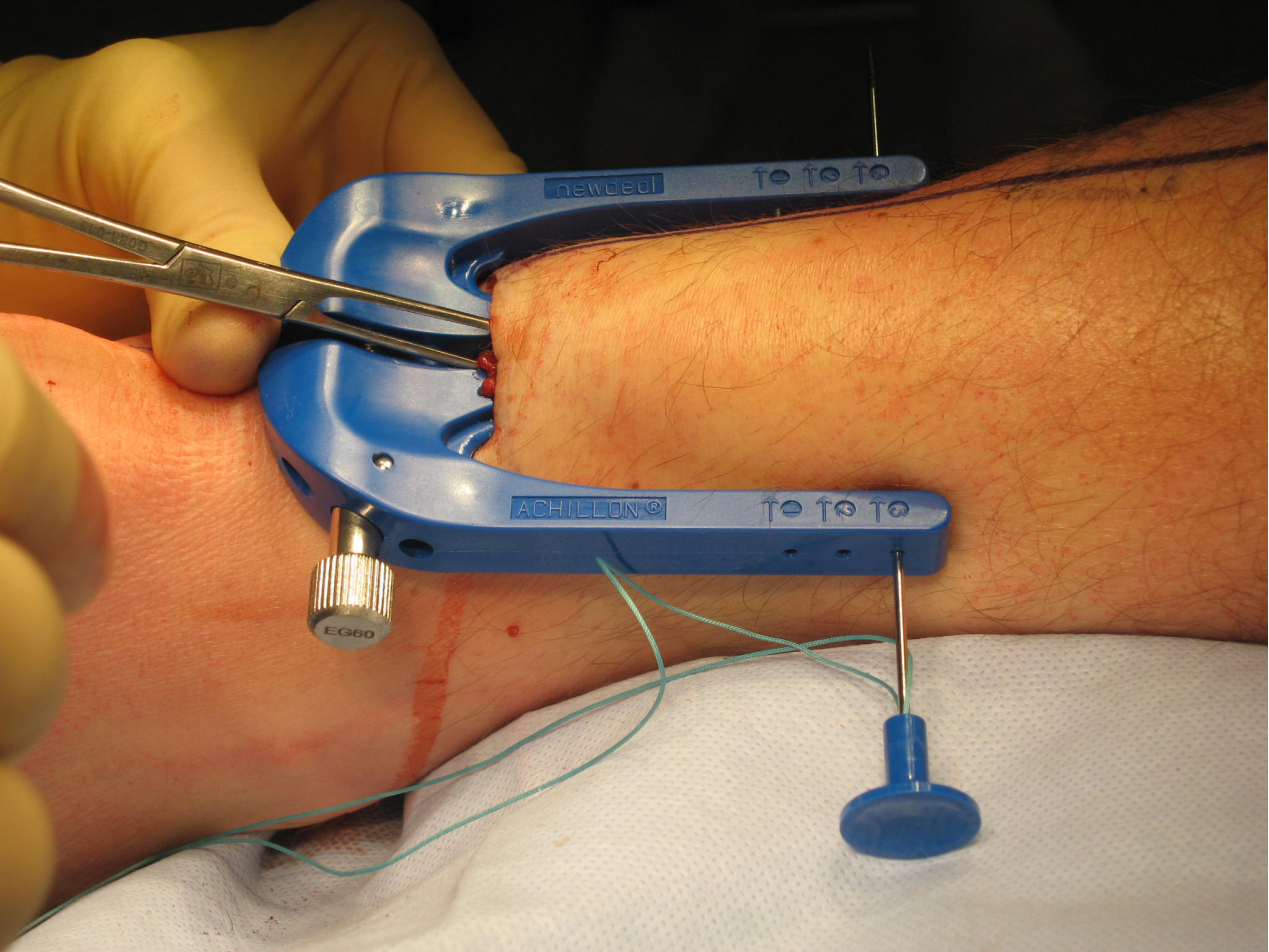
**Sutures are passed through skin, subcutaneous fat fascia, cruris, paratenon and tendon**.

**Figure 7:**
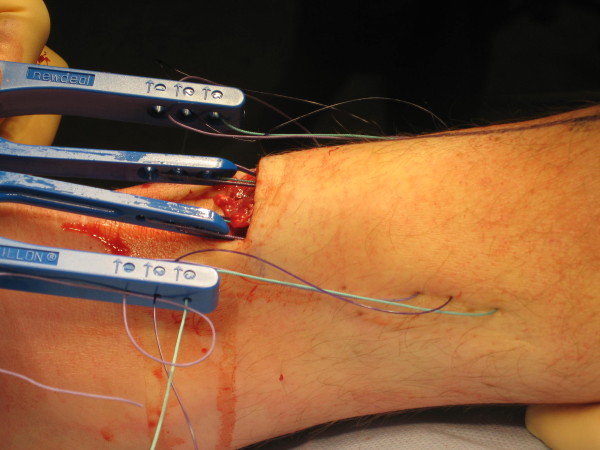
**When the jig is withdrawn the three sutures are left within the paratenon and traverse the mid-portion of the tendon**.

The Achillon jig (Integra Lifesciences Corporation, USA) has been developed from an initial minimally invasive technique [41,42]. Transtendinous sutures can be placed through targeting holes in a jig across the two layers of the skin, fascia cruris, paratenon and the proximal Achilles [43] (Figures 5 &6). When the jig is removed the tendon traversing sutures remain within the paratenon (Figure 7) and so they can be tied within the paratenon opposing the tendon ends. Clearly there is a risk of nerve puncture but as the sutures are removed through the same puncture pass the risk of permanent nerve symptoms are very unlikely but possible.

The designers of the jig reported the outcome of 68 patients followed up for 26 months. The mean AOFAS score was 96, there were no wound healing problems or sensory disturbance. There were two cases of re-rupture associated with poor compliance to rehabilitation [43].

The use of the jig has been independently evaluated in a series of 25 patients using early active rehabilitation. Return to sporting activity occurred after 5 months and there were no wound problems, sural nerve injuries or re-ruptures [44].

The strength of the repair technique has been compared to a two-strand Kessler technique both using Number 2 Tycron on Ovine Achilles tendons. The 6 strand Achillon repair had similar strength (153N load to failure) to that of the Kessler repair (123N load to failure, p = 0.21) [45].

In a small series of 15 patients repaired using the jig, Chan reported a recovery of 95% isometric peak force recovery compared to the non-injured side, resumption of sports activity and no complications [46].

In a case control study using a percutaneous technique the sural nerve was injured in 18% of repairs in which the nerve was not exposed intra-operatively [47]. It is recommended that the sural nerve is visualised during repair to minimise injury [47,48].

Metz and co-workers have considered the frequency and severity of complications by surveying patients who had a ruptured Achilles tendon treated by minimally invasive repair [49]. The technique used was that described by Bijlsma, consisting of a Bunnel polydioxanone suture placed in the proximal end of the Achilles tendon through a small longitudinal incision. The suture is then passed through a drill hole in the calcaneus after subcutaneous tunnelling and tied with the ankle in relaxed equinus position [50]. Two hundred and eleven patients returned questionnaires. There was an overall complication rate of 36%. The mean ATRS was 84 at a mean follow up of 6.2 years. There were 135 uncomplicated cases for whom the ATRS was 89, whereas for those with complications ATRS was lower. For the 8% with re-rupture ATRS was 71, the 19% with sural nerve injury the score was 75. Minor wound problems did not really lower the score however the patient with a severe wound infection had a very low score of 28 points.

### Comparative studies, percutaneous/minimally invasive versus open

Over the past 10 years there are a number of series, which compare minimally invasive techniques with open repairs. It is clear that different techniques have been compared over the same period of time as a result direct comparison is difficult on account of varying techniques and outcome measures.

Cetti compared 111 Achilles tendon ruptures randomized prospectively to operative and non-operative treatment. Those treated operatively had a higher rate of resumption of sporting activity at the same level, less calf atrophy, better ankle movement and fewer complaints than those treated non operatively. There were 2 deep infections in the operative group and 3 re-ruptures compared to 7 re-ruptures in the non-operative group. One patient managed non-operatively had a second re-rupture [51].

Lim reported the outcome of Ma & Griffith percutaneous technique and an open repair consisting of a Kessler suture supplemented with interrupted sutures in a prospective randomised multicentre study. It was notable that 7 patients had paraesthesia in the sural nerve distribution prior to surgery. There was a significant difference in wound complications, with increased wound infections in open repairs (21%). Percutaneous repair was advocated on the low rate of complications and improved cosmetic appearance [52].

In 2002 Riedl reported a series performed from 1992-1999. There were complications in 6% of patients who had a percutaneous suture performed compared to 18% of those who had open repair. There were some disturbances in sensitivity of the sural nerve after percutaneous repair, notably there was a 6% incidence in thromboembolism reported in the open group [53].

Haji retrospectively compared percutaneous and open repair techniques. There were 70 open repairs compared with 38 repairs using the Ma & Griffiths technique. The percutaneous method took a considerably shorter period of time 28.5 minutes than the open repairs 45 minutes. There were 4 cases of re-rupture (5.7%), 4 deep infections (4.7%) and two palpable suture knots (2.9%) in the open group. In the percutaneous group there was only one re-rupture, five palpable suture knots (13.2%) and four transient nerve lesions (10.5%) but no infections [54].

Wagnon and Akayi compared percutaneous using the Webb-Bannister technique versus open repair of the Achilles tendon over an 8 year period. Clinical and functional results were similar although there was an 8.6% incidence of wound complications in the open group. Return to work times were not significantly different 4 months for open repair and 3.75 months for percutaneous repair [55].

Cretnik has compared 132 patients using a percutaneous repair under local anaesthetic with 100 open procedures. Overall there were less complications in the percutaneous group 9.7% vs. 21% in the open group. There were slightly more re-ruptures (3.7%) and sural nerve disturbances (4.5%) in the percutaneous group [56].

A prospective comparison study in 40 patients between open and percutaneous repair using the Tenolig showed similar outcome parameters for both groups; SF12, ultrasound and isokinetic testing. There were two minor complications in the open group and one failed repair in the percutaneous group [57].

Metz has performed a randomized controlled trial comparing minimally invasive repair versus non-operative treatment. Complication risk other than re-rupture on an intention to treat basis was 21% for surgical treatment and 36% for non-operative treatment. Patients returned to work significantly sooner for operative repair (59 days) compared to non-operative (108 days) [58].

When minimally invasive repairs using the Achillon device were compared with open repairs over a two-year period, Aktas reported similar AOFAS outcome scores in both groups. There was a significantly better outcome regarding tenderness, skin adhesions, scar and tendon thickness in those repaired using the minimally invasive technique [59].

Bhattacharyya and Gerber also compared minimally invasive repair using the Achillon technique through a 3 cm incision medial to the palpable tendon gap with early weight bearing versus open repairs with weight bearing at 8 weeks of the Achilles tendon. There was a significant number of wound infections and wound pain in the open repair patients [60]. The authors noted that there was less in patient bed occupancy and analgesic requirement particularly opiates in the open group (mean 3.3 days vs. 1 day).

Henriquez suggested is a retrospective study that percutaneous repair provided similar function to that with open repair, with a better cosmetic appearance, a lower rate of wound complications and no apparent increase in the risk of re-rupture. The mean time taken to return to work was longer for open repair 5.6 months compared to percutaneous 2.8 months [61].

Khan has recently completed a meta-analysis of surgical intervention for Achilles tendon repair methods. Open surgical treatment had a higher risk of infection (RR 4.89), adhesions and disturbed skin sensibility [7].

In summary these series report similar functional outcome in both series. Although those treated with percutaneous and minimally invasive repairs had slightly higher rates of re-rupture and sural nerve damage, hospital stay, wound complications and return to work were all considerably lower.

The major disadvantage of percutaneous repair is that it may not be possible to ensure that the ruptured tendon ends have been apposed by the repair. With the increased use of endoscopy with hindfoot procedures and intra-operative ultrasound these modalities permit the indirect visualisation of the repair.

### Endoscopic assistance

Turgut first described a series of percutaneous Achilles repair performed with endoscopic assistance following cadaveric a study [62]. This technique was used in eleven patients all of whom returned to daily activities at 10-11 weeks post repair. There were no re-ruptures or nerve injuries.

Halasi subsequently used endoscopy to assist percutaneous Achilles repair in 67 cases of a large series of 156 Achilles repairs. Results were excellent or good in 88% of both groups of patients. The re-rupture rate was found to be lower in the endoscopy controlled group with only 1 re-rupture compared to 5 re-ruptures in the percutaneous group [63].

Tang reported the use of arthroscopic assistance to perform percutaneous repair of the Achilles tendon. The torn ends of the Achilles were debrided using arthroscopy and then a percutaneous repair of the Achilles was performed using a Kessler suture by an inside out technique [64]. According to the Lindholm scale all patients had excellent or good scores and no patients reported nerve injury infections or re-rupture at follow up [65].

Endoscopic control has been utilised to allow a Bunnel method to repair the debrided tendon ends in 62 patients [66]. There were no significant range of movement limitations and satisfactory AOFAS scores were obtained (94.6 at 46 months mean follow up). Only two patients experienced transient sural nerve hypothesia resolving within 6 months. Return to regular work and rehabilitation training occurred at 11.7 weeks following surgery and no re-ruptures of wound problems were reported.

A series of 20 patients had a percutaneous repair performed with endoscopic assistance and where followed up at 2.5 years, all reporting excellent/good outcomes. Two patients reported sural neuralgia however there were no wound problems, re-ruptures or infections reported [67].

It is worthy of note the Lui has described an endoscopic assisted repair technique for repair of the Achilles tendon however describes the technique as complicated and it required 6 portals [68]. A cadaveric study identified that suture placement was an issue and it was important to prevent the suture from falling into the tendon gap. Grasping of the suture with Allis forceps also aids suture placement [69]. He has adapted a technique for the reattachment of the Achilles tendon for insertional tendinopathy to the repair of ruptures. This involves placing a locking Krakow suture through a 1.5 cm medial incision [70].

Clearly there is a considerable learning curve associated with endoscopic assistance and it is likely to involved increased operating time. Nerve injury rates do not appear to be increased. Another potential disadvantage is the washing away of the haematoma by the irrigation fluid. The loss of the valuable components of the haematoma could be thought to weaken the repair, although increased re-rupture rates were not noted.

### Ultrasound assistance

One of the advantages of open repair methods over percutaneous repair is the ability to actually see that the tendon ends are opposed. The use of ultrasound allows confirmation of the absence of gaping in both the post-operative period and intra-operatively. Accurate imaging with ultrasound requires considerable skill and may require many years of practice.

Real time ultrasonography has been used in combination percutaneous Achilles repair [71] in a series of 5 patients using Ma & Griffiths technique. The authors reported good visualisation of tendon approximation in every case when US was used. In the same report an additional 15 patients had immediate post-operative ultrasound performed. They used the post-operative imaging findings at 3 weeks to guide weight bearing. Those with incomplete healing received non-weight bearing and gentle physiotherapy, whereas those with good healing were permitted to full weight bearing and received intensive physiotherapy.

The effectiveness of ultrasound as an intra-operative tool during percutaneous repair was shown in a study of the placement of intra-tendinous needles. Whereas only 55% of needles were correctly positioned without imaging, all needles placed using ultrasound showed correct positioning and the ultrasound confirmed stump approximation [72].

### Biological Augmentation

The use of biological materials such as Platelet Rich Plasma, to promote healing is an exciting and controversial development relevant to Achilles tendon surgery. To date these techniques have only been applied to open tendon repairs. Sanchez reported earlier range of motion, less time to gentle running and resumption of training in a case control study in which a platelet rich fibrin matrix was applied to the sutured tendon before closure [73]. Other randomised controlled studies using a different preparation method of PRP, have failed to show a difference in outcome [74]. Randomised controlled studies consisting of standardised preparation methods and application techniques are required to determine the efficacy of PRP. Given that proponents of minimally invasive surgery preserve soft tissue to enhance healing it is likely that these surgeons will utilise biological augmentation to promote recovery. Results are awaited.

### Post-operative rehabilitation

The developments of surgery through percutaneous and minimally invasive incisions have gone hand in hand with those of rehabilitation following repair. The key principles here are of immediate weight bearing and early functional bracing and rehabilitation.

Studies have shown that immediate post-operative weight bearing is not detrimental to outcome [75]. Costa has shown no evidence of tendon lengthening or increased re-rupture rate with immediate weight bearing following surgical repair [76]. Costa also reported no detriment from immediate weight bearing mobilisation and advocates immediate weight bearing as a result of the two studies [77].

Calder in rehabilitation of repairs performed using the Achillon jig maintained patients at 20 degrees of plantar flexion for 2 weeks post -operatively and then permitted them to move from neutral to full plantar flexion for 4 weeks. There were no re-ruptures and all patients were able to return to full sporting activity by 6 months following surgery [78].

The use of functional braces rather than plaster casts are now common place. Early functional therapy with a special shoe lead to significantly earlier return to work (37 days) compared to cast immobilisation (67 days) (0.042.) in a retrospective case control study [79].

It is worthy of note that Twaddle reported on a randomized prospective study between surgically and non-surgically treated Achilles tendon ruptures. Patients all received early motion controlled in an orthosis and progressed to full weight bearing at 8 weeks from treatment. There were no significant differences in outcome scores, complications or re-ruptures in each group suggesting that controlled early motion is an important part of treatment [80].

### Neglected rupture

As experience and understanding of minimally invasive techniques progresses the next stage is the advancement of these methods during reconstructive surgery following Achilles injury. Bertelli reports on a series of 20 patients in which percutaneous suturing of the Achilles tendon is used for neglected rupture using 10 micro-incisions with a number 1 Vicryl suture in a figure of 8 fashion [81].

## Summary

Percutaneous and minimally invasive Achilles repair offers good outcome following Achilles tendon rupture, with less wound complications, hospital stay and earlier return to work. There is however a small increase in risk of iatrogenic sural nerve injury.

We recommend careful examination following injury for sural neuropraxia pre surgery and visualisation of the nerve during repair. Direct apposition of the tendon ends rather than pure restoration of the tenodesis effect of the tendon is beneficial. This can be achieved with direct vision, endoscopy or intra-operative ultrasound.

Post operatively immediate weight bearing and rehabilitation incorporating early movement.

## Competing interests

The authors declare that there are no competing interests.

## Authors' contributions

MC, RR, SS, OMD and PB all contributed to the writing of this review paper. All authors have read and approved the final manuscript.
